# Supplementary material for: Guinea Pig X Virus Is a Gammaherpesvirus
Source: Viruses. 2025 Aug 5;17(8):1084. doi: 10.3390/v17081084 (PMC12390642; doi:10.3390/v17081084)
Supplement: Supplementary file 1 [file viruses-17-01084-s001.zip › viruses-3753675-supplementary/Supplementary Table 1 Viruses Used for Phylogenetic Comparison with GPXV.pdf]

## Supplementary Table S1: Viruses Used for Phylogenetic Comparison with GPXV

| Common Name | ICTV Formal Name | Natural Host | Accession Number (NCBI) |
|---|---|---|---|
| ORF8 Guinea Pig X Virus | Guinea Pig X Virus | Guinea pigs (Cavia porcellus) | PV335591.1 |
| BALF4-like glycoprotein Marmot herpesvirus 1 | Marmot herpesvirus 1 | Marmot species | UNP64606.1 |
| JM12 Macaca fuscata rhadinovirus | Macaca fuscata rhadinovirus | Japanese macaque (Macaca fuscata) | YP_238315.1 |
| Glycoprotein B Macacine gammaherpesvirus 5 | Macacine gammaherpesvirus 5 | Rhesus macaque (Macaca mulatta) | NP_570749.1 |
| ORF8 Rhesus monkey rhadinovirus H26-95 | Rhesus monkey rhadinovirus H26-95 | Rhesus macaque (Macaca mulatta) | AAF59985.1 |
| Glycoprotein B Rhesus monkey rhadinovirus H26-95 | Rhesus monkey rhadinovirus H26-95 | Rhesus macaque (Macaca mulatta) | AAC58686.1 |
| Glycoprotein gB Macaca mulatta rhadinovirus | Macaca mulatta rhadinovirus | Rhesus macaque (Macaca mulatta) | ADB08238.1 |
| ORF8 Macacine gammaherpesvirus 5 | Macacine gammaherpesvirus 5 | Rhesus macaque (Macaca mulatta) | QFQ66777.1 |
| Glycoprotein B Retroperitoneal fibromatosis-associated herpesvirus | Retroperitoneal fibromatosis-associated herpesvirus | Rhesus macaque (Macaca mulatta) | AAF81661.2 |
| ORF8 Human gammaherpesvirus 8 | Human gammaherpesvirus 8 | Humans (Homo sapiens) | QKE51418.1 |
| Envelope glycoprotein gB Human gammaherpesvirus 8 | Human gammaherpesvirus 8 | Humans (Homo sapiens) | ADB08179.1 |
| ORF9 Guinea Pig X Virus | Guinea Pig X Virus | Guinea pigs (Cavia porcellus) | PV335591.1 |
| Polymerase subunit Marmot herpesvirus 1 | Marmot herpesvirus 1 | Marmot species | UNP64605.1 |
| DNA polymerase catalytic subunit Peromyscus leucopus gammaherpesvirus | Peromyscus leucopus gammaherpesvirus | White-footed mouse (Peromyscus leucopus) | WIM51710.1 |
| Polymerase subunit Saguinine gammaherpesvirus 1 | Saguinine gammaherpesvirus 1 | Common marmoset (Callithrix jacchus) | UNP64511.1 |
| JM14 Macaca fuscata rhadinovirus | Macaca fuscata rhadinovirus | Japanese macaque (Macaca fuscata) | YP_238317.1 |

| | | | |
|---|---|---|---|
| ORF9 Human gammaherpesvirus 8 | Human gammaherpesvirus 8 | Humans (Homo sapiens) | XCA92092.1 |
| DNA polymerase Myotis ricketti herpesvirus 1 | Myotis ricketti herpesvirus 1 | Rickett's big-footed bat (Myotis ricketti) | AFM85234.1 |
| DNA polymerase Saimiriine gammaherpesvirus 2 | Saimiriine gammaherpesvirus 2 | Squirrel monkey (Saimiri sciureus) | NP_040211.1 |
| DNA polymerase Bovine gammaherpesvirus 4 | Bovine gammaherpesvirus 4 | Cattle (Bos taurus) | WIV69218.1 |
| DNA polymerase catalytic subunit Rhinolophus gammaherpesvirus 1 | Rhinolophus gammaherpesvirus 1 | Horseshoe bats (Rhinolophus spp.) | YP_009551819.1 |
| DNA polymerase catalytic subunit Myotis gammaherpesvirus 8 | Myotis gammaherpesvirus 8 | Mouse-eared bats (Myotis spp.) | YP_009229846.1 |
| Protein Rta Myotis gammaherpesvirus 8 | Myotis gammaherpesvirus 8 | Mouse-eared bats (Myotis spp.) | YP_009229881.1 |
| Protein Rta Macronycteris gammaherpesvirus 1 | Macronycteris gammaherpesvirus 1 | Large-eared free-tailed bat (Macronycteris spp.) | BEG23121.1 |
| Protein Rta Rhinolophus gammaherpesvirus 1 | Rhinolophus gammaherpesvirus 1 | Horseshoe bats (Rhinolophus spp.) | YP_009551858.1 |
| R transactivator protein Bovine gammaherpesvirus 4 | Bovine gammaherpesvirus 4 | Cattle (Bos taurus) | WIV69221.1 |
| Transcription activation factor Sea otter herpesvirus | Sea otter herpesvirus | Sea otter (Enhydra lutris) | AOT85981.1 |
| Transcription activator factor Eptesicus fuscus gammaherpesvirus | Eptesicus fuscus gammaherpesvirus | Big brown bat (Eptesicus fuscus) | YP_009552512.1 |
| ORF50 Murid gammaherpesvirus 4 | Murid gammaherpesvirus 4 | Mice (Mus musculus) | AAF19314.1 |
| Protein Rta Wood mouse herpesvirus | Wood mouse herpesvirus | Wood mouse (Apodemus sylvaticus) | YP_010085924.1 |
| ORF50 Guinea Pig X Virus | Guinea Pig X Virus | Guinea pigs (Cavia porcellus) | PV335591.1 |
| Putative herpesvirus transcription activation factor Marmot herpesvirus 1 | Marmot herpesvirus 1 | Marmot species | UNP64563.1 |
| Putative herpesvirus transcription activation | Saguinine gammaherpesvirus 1 | Common marmoset (Callithrix jacchus) | UNP64472.1 |

| | | | |
|---|---|---|---|
| factor Saguinine gammaherpesvirus 1 | | | |
| Hypothetical protein MRV_0112 Murine roseolovirus | Murine roseolovirus | Mice (Mus musculus) | YP_009344937.1 |
| ORF73 Macaca nemestrina rhadinovirus 2 | Macaca nemestrina rhadinovirus 2 | Pig-tailed macaque (Macaca nemestrina) | YP_010084631.1 |
| Latency-associated nuclear antigen Rhesus monkey rhadinovirus H26-95 | Rhesus monkey rhadinovirus H26-95 | Rhesus macaque (Macaca mulatta) | AAF60071.1 |
| Latent nuclear antigen Macacine gammaherpesvirus 5 | Macacine gammaherpesvirus 5 | Rhesus macaque (Macaca mulatta) | NP_570820.1 |
| ORF73 Macacine gammaherpesvirus 5 | Macacine gammaherpesvirus 5 | Rhesus macaque (Macaca mulatta) | QFQ66859.1 |
| ORF73 Felid gammaherpesvirus 1 | Felid gammaherpesvirus 1 | Domestic cat (Felis catus) | YP_009173954.1 |
| ORF73 Guinea Pig X Virus | Guinea Pig X Virus | Guinea pigs (Cavia porcellus) | PV335591.1 |
| ORF73 Human gammaherpesvirus 8 | Human gammaherpesvirus 8 | Humans (Homo sapiens) | QLI54804.2 |

**Table 1.** List of viruses used for phylogenetic comparison with Guinea Pig X Virus (GPXV), including their ICTV formal names, common names, natural hosts and accession numbers.
