# Supplementary material for: Guinea Pig X Virus Is a Gammaherpesvirus
Source: Viruses. 2025 Aug 5;17(8):1084. doi: 10.3390/v17081084 (PMC12390642; doi:10.3390/v17081084)
Supplement: Supplementary file 1 [file viruses-17-01084-s001.zip › viruses-3753675-supplementary/Supplementary Table 2 Annotated GPXV Genes with Coordinates.pdf]

Supplementary Table S2: Annotated GPXV Genes with Coordinates

Gene	Start	End	Strand
G1	124	1410	-
G2	1547	1726	-
G3	2019	3314	+
G4	3517	3663	-
ORF4	3665	4783	+
ORF6	5363	8374	+
ORF7	8374	10452	+
ORF8	10442	12979	+
ORF9	13234	16221	+
G5	16294	16455	+
ORF10	16424	17671	+
K3	17706	18284	-
G6	18244	18351	-
G7	18883	19104	+
G8	19602	20222	-
ORF17.5	20503	21342	-
G9	22498	23016	+
ORF17	23051	23998	-
ORF18	24018	24776	+
ORF19	24790	26178	-
ORF20	26150	26854	-
ORF21	26943	28577	+
ORF22	28561	30735	+
ORF23	30732	31892	-
ORF24	31889	34027	-
ORF25	34050	38171	+
ORF26	38190	39092	+
ORF27	39077	39976	+
ORF29	40011	41150	-
ORF30	41151	41393	+
ORF31	41351	41956	+
ORF32	41941	43257	+
ORF33	43250	44236	+
ORF29a	44130	45125	-
ORF34	45130	46086	+
ORF35	46073	46543	+
ORF36	46578	47732	+
ORF37	47752	49200	+
ORF38	49155	49397	+
ORF39	49583	50710	-
ORF40	50823	52208	+
ORF41	52308	52820	+
ORF42	52812	53627	-
ORF43	53569	55299	-

ORF44	55301	57592	+
ORF45	57665	58255	-
ORF46	58282	59040	-
G10	59042	59206	-
G11	59217	59468	+
ORF48	59523	60974	-
ORF49	61175	62068	-
ORF50	62167	63912	+
G12	63974	64069	+
G13	64084	64329	+
G14	64561	64869	+
ORF52	64975	65349	-
ORF53	65410	66423	-
ORF54	66492	67427	+
ORF55	67497	68063	-
ORF56	68111	70639	+
ORF5	70899	72278	+
G15	72506	72616	+
ORF58	72705	73769	-
ORF59	73772	74941	-
ORF60	75081	75998	-
ORF61	76021	78333	-
ORF62	78335	79327	-
ORF63	79329	82097	+
ORF64	82101	89753	+
ORF65	89805	90233	-
ORF66	90240	91469	-
ORF67A1	91412	92191	-
ORF67A2	92178	92447	-
ORF68	92624	93985	+
ORF69	93997	94902	+
G16	95075	95203	+
ORF72	97253	97963	-
ORF16	98169	98711	-
ORF73	98722	100311	-
G17	100931	101215	+
ORF74	101460	102491	+
ORF75	102621	106499	-
G18	106634	107167	-
G19	107971	108132	+
G20	108362	108859	-

Table 2. Coordinates and strand orientation of predicted genes in the Guinea Pig X Virus (GPXV) genome. Start and end positions are based on the annotated GPXV genome to NCBI. Strand direction was inferred from coordinate orientation, where genes with decreasing coordinates are located on the reverse (−) strand.
