# Supplementary material for: Guinea Pig X Virus Is a Gammaherpesvirus
Source: Viruses. 2025 Aug 5;17(8):1084. doi: 10.3390/v17081084 (PMC12390642; doi:10.3390/v17081084)
Supplement: Supplementary file 1 [file viruses-17-01084-s001.zip › viruses-3753675-supplementary/Supplementary Table 3 Genomic Coordinates of Selected ORFs in GPXV.pdf]

Supplementary Table S3: Genomic Coordinates of Selected ORFs in GPXV

ORF(s)	Start Position	End Position	Strand
ORF8	10442	12979	+
ORF9	13234	16221	+
ORF50	62167	63912	+
ORF73	98722	100311	_

Table 3. Genomic coordinates of selected open reading frames (ORFs) 8, 9, 50, and 73 in the GPXV genome. The table lists the ORF names, their corresponding start and end positions, and the DNA strand orientation.
