# Supplementary material for: Guinea Pig X Virus Is a Gammaherpesvirus
Source: Viruses. 2025 Aug 5;17(8):1084. doi: 10.3390/v17081084 (PMC12390642; doi:10.3390/v17081084)
Supplement: Supplementary file 1 [file viruses-17-01084-s001.zip › viruses-3753675-supplementary/Supplementary Table 4 GPXV and GPHLV Comparision.pdf]

GPXV Protein/Gene	GPHLV Protein/Gene	GPXV Start	GPXV End	GPHLV Start	GPHLV End	% Identity	Notes
3_15	—	123.0	1410.0	—	—	—	Unique to GPXV
GPXV Unknown Protein	—	123.0	1410.0	—	—	—	Unique to GPXV
3_14	—	1546.0	1726.0	—	—	—	Unique to GPXV
3_13	—	2018.0	3314.0	—	—	—	Unique to GPXV
K1 positional homologue	K12 positional homolog	2018.0	3314.0	91850.0	91952.0	76.0	Divergent
3_12	—	3516.0	3663.0	—	—	—	Unique to GPXV
ORF4	ORF40	3664.0	4783.0	48482.0	50375.0	81.25	Divergent
ORF6	ORF6	5362.0	8374.0	3526.0	6865.0	80.0	Divergent
ORF7	ORF7	8373.0	10452.0	6867.0	8901.0	88.89	Divergent
ORF8	ORF8	10441.0	12979.0	8902.0	11464.0	83.78	Divergent
Glycoprotein B	—	10441.0	12979.0	—	—	—	Unique to GPXV
ORF9	ORF9	13233.0	16221.0	11577.0	14553.0	88.89	Divergent
3_6	—	16293.0	16455.0	—	—	—	Unique to GPXV
ORF10	ORF10	16423.0	17671.0	14706.0	15972.0	85.71	Divergent
K3	K3	17705.0	18284.0	16012.0	16576.0	86.67	Divergent
3_3	—	18243.0	18351.0	—	—	—	Unique to GPXV
3_2	—	18882.0	19104.0	—	—	—	Unique to GPXV
CDS_19601_20222	—	19601.0	20222.0	—	—	—	Unique to GPXV
ORF17.5	ORF17	20502.0	21342.0	20103.0	21675.0	76.92	Divergent
vIL10	—	22497.0	23016.0	—	—	—	Unique to GPXV
ORF17	ORF17	23050.0	23998.0	20103.0	21675.0	86.96	Divergent
ORF18	ORF18	24017.0	24776.0	21661.0	22441.0	86.96	Divergent
ORF19	ORF19	24789.0	26178.0	22453.0	24082.0	78.57	Divergent
ORF20	ORF10	26149.0	26854.0	14706.0	15972.0	78.57	Divergent
ORF21	ORF21	26942.0	28577.0	24609.0	26262.0	87.23	Divergent
ORF22	ORF22	28560.0	30735.0	26245.0	28432.0	84.62	Divergent
ORF23	ORF23	30731.0	31892.0	28417.0	29617.0	87.76	Divergent
ORF24	ORF29	31888.0	34027.0	17979.0	18225.0	80.65	Divergent
ORF25	ORF25	34049.0	38171.0	31782.0	35907.0	90.0	Divergent

GPXV Protein/Gene	GPHLV Protein/Gene	GPXV Start	GPXV End	GPHLV Start	GPHLV End	% Identity	Notes
ORF26	ORF26	38189.0	39092.0	35927.0	36833.0	89.83	Divergent
ORF27	ORF27	39076.0	39976.0	36817.0	37708.0	86.05	Divergent
ORF29	ORF29	40010.0	41150.0	17979.0	18225.0	79.31	Divergent
ORF30	ORF30	41150.0	41393.0	38870.0	39113.0	60.0	Divergent
ORF31	ORF10	41350.0	41956.0	14706.0	15972.0	82.35	Divergent
ORF32	ORF32	41940.0	43257.0	39654.0	40986.0	87.23	Divergent
ORF33	ORF23	43249.0	44236.0	28417.0	29617.0	82.86	Divergent
ORF29a	ORF29a	44129.0	45125.0	41837.0	42860.0	86.67	Divergent
ORF34	ORF23	45129.0	46086.0	28417.0	29617.0	81.82	Divergent
ORF35	ORF35	46072.0	46543.0	43792.0	44263.0	63.46	Divergent
ORF36	ORF6	46577.0	47732.0	3526.0	6865.0	82.35	Divergent
ORF37	ORF37	47751.0	49200.0	45458.0	46907.0	73.03	Divergent
ORF38	ORF8	49154.0	49397.0	8902.0	11464.0	71.43	Divergent
ORF39	ORF39	49582.0	50710.0	47252.0	48377.0	91.67	Highly conserved
ORF40	ORF40	50822.0	52208.0	48482.0	50375.0	87.23	Divergent
ORF41	ORF10	52307.0	52820.0	14706.0	15972.0	81.82	Divergent
ORF42	ORF42	52811.0	53627.0	50366.0	51188.0	86.67	Divergent
ORF43	ORF43	53568.0	55299.0	51126.0	52863.0	90.62	Highly conserved
ORF44	ORF44	55300.0	57592.0	52942.0	55165.0	86.67	Divergent
ORF45	ORF25	57664.0	58255.0	31782.0	35907.0	76.92	Divergent
ORF46	ORF46	58281.0	59040.0	55788.0	56547.0	69.84	Divergent
1_15	—	59041.0	59206.0	—	—	—	Unique to GPXV
1_14	—	59216.0	59468.0	—	—	—	Unique to GPXV
ORF48	ORF48	59522.0	60974.0	57033.0	58572.0	86.96	Divergent
ORF49	ORF49	61174.0	62068.0	58770.0	59661.0	88.46	Divergent
ORF50	ORF50	62166.0	63912.0	59758.0	61558.0	89.09	Divergent
1_10	—	63973.0	64069.0	—	—	—	Unique to GPXV
1_9	—	64083.0	64329.0	—	—	—	Unique to GPXV
1_8	—	64560.0	64869.0	—	—	—	Unique to GPXV
ORF52	ORF52	64974.0	65349.0	62556.0	62931.0	19.35	Divergent
ORF53	ORF53	65409.0	66423.0	63003.0	63738.0	83.78	Divergent
ORF54	ORF54	66491.0	67427.0	63819.0	64758.0	87.23	Divergent
ORF55	ORF25	67496.0	68063.0	31782.0	35907.0	73.91	Divergent

GPXV Protein/Gene	GPHLV Protein/Gene	GPXV Start	GPXV End	GPHLV Start	GPHLV End	% Identity	Notes
ORF56	ORF56	68110.0	70639.0	65420.0	67955.0	90.0	Divergent
ORF57	ORF57	70898.0	72278.0	68268.0	69579.0	82.35	Divergent
1_1	—	72505.0	72616.0	—	—	—	Unique to GPXV
ORF58	ORF58	72704.0	73769.0	69881.0	70946.0	64.41	Divergent
ORF59	ORF59	73771.0	74941.0	70948.0	72046.0	89.09	Divergent
ORF60	ORF60	75080.0	75998.0	72157.0	73075.0	79.34	Divergent
ORF61	ORF61	76020.0	78333.0	73095.0	75393.0	88.46	Divergent
ORF62	ORF6	78334.0	79327.0	3526.0	6865.0	80.0	Divergent
ORF63	ORF63	79328.0	82097.0	76383.0	79134.0	88.0	Divergent
ORF64	ORF64	82100.0	89753.0	79136.0	86927.0	89.29	Divergent
ORF65	ORF65	89804.0	90233.0	87018.0	87423.0	88.68	Divergent
ORF66	ORF66	90239.0	91469.0	87412.0	88663.0	82.35	Divergent
ORF67A	ORF67A	92177.0	92447.0	89338.0	89623.0	89.83	Divergent
ORF68	ORF68	92623.0	93985.0	89733.0	91092.0	87.5	Divergent
ORF69	ORF69	93996.0	94902.0	91095.0	92010.0	85.37	Divergent
2_1	—	95074.0	95203.0	—	—	—	Unique to GPXV
ORF72	ORF72	97252.0	97963.0	93856.0	94570.0	86.67	Divergent
ORF16	ORF16	98168.0	98711.0	94762.0	95293.0	82.35	Divergent
LANA	LANA	98721.0	100311.0	95292.0	96945.0	83.78	Divergent
CDS_100930_101215	—	100930.0	101215.0	—	—	—	Unique to GPXV
CDS_101215_101221	—	101215.0	101221.0	—	—	—	Unique to GPXV
CDS_101221_101314	—	101221.0	101314.0	—	—	—	Unique to GPXV
ORF74	ORF74	101459.0	102491.0	97890.0	98955.0	82.86	Divergent
ORF75	ORF75	102620.0	106499.0	98999.0	102869.0	86.05	Divergent
4_4	—	106633.0	107167.0	—	—	—	Unique to GPXV
4_5	—	107970.0	108132.0	—	—	—	Unique to GPXV
4_6	—	108361.0	108859.0	—	—	—	Unique to GPXV
—	G12	—	—	0.0	111.0	—	Unique to GPHLV
—	TR 2	—	—	0.0	111.0	—	Unique to GPHLV
—	CDS_197_1394	—	—	197.0	1394.0	—	Unique to GPHLV

GPXV Protein/Gene	GPHLV Protein/Gene	GPXV Start	GPXV End	GPHLV Start	GPHLV End	% Identity	Notes
—	CDS_2185_3313	—	—	2185.0	3313.0	—	Unique to GPHLV
—	CDS_3526_6865	—	—	3526.0	6865.0	—	Unique to GPHLV
—	CDS_6867_8901	—	—	6867.0	8901.0	—	Unique to GPHLV
—	CDS_8902_11464	—	—	8902.0	11464.0	—	Unique to GPHLV
—	CDS_11577_14553	—	—	11577.0	14553.0	—	Unique to GPHLV
—	K1	—	—	15813.0	15984.0	—	Unique to GPHLV
—	CDS_16012_16576	—	—	16012.0	16576.0	—	Unique to GPHLV
—	G10	—	—	17356.0	17629.0	—	Unique to GPHLV
—	CDS_17356_17629	—	—	17356.0	17629.0	—	Unique to GPHLV
—	G9	—	—	17979.0	18225.0	—	Unique to GPHLV
—	G8	—	—	18397.0	18544.0	—	Unique to GPHLV
—	G7	—	—	18540.0	18654.0	—	Unique to GPHLV
—	CDS_20103_21675	—	—	20103.0	21675.0	—	Unique to GPHLV
—	CDS_22453_24082	—	—	22453.0	24082.0	—	Unique to GPHLV
—	CDS_23768_24611	—	—	23768.0	24611.0	—	Unique to GPHLV
—	CDS_24609_26262	—	—	24609.0	26262.0	—	Unique to GPHLV
—	CDS_26245_28432	—	—	26245.0	28432.0	—	Unique to GPHLV
—	CDS_28417_29617	—	—	28417.0	29617.0	—	Unique to GPHLV
—	CDS_29627_31784	—	—	29627.0	31784.0	—	Unique to GPHLV
—	CDS_31782_35907	—	—	31782.0	35907.0	—	Unique to GPHLV
—	CDS_35927_36833	—	—	35927.0	36833.0	—	Unique to GPHLV

GPXV Protein/Gene	GPHLV Protein/Gene	GPXV Start	GPXV End	GPHLV Start	GPHLV End	% Identity	Notes
—	CDS_36817_37708	—	—	36817.0	37708.0	—	Unique to GPHLV
—	ORF29b	—	—	37725.0	38775.0	—	Unique to GPHLV
—	CDS_37725_38775	—	—	37725.0	38775.0	—	Unique to GPHLV
—	CDS_38870_39113	—	—	38870.0	39113.0	—	Unique to GPHLV
—	CDS_39070_39670	—	—	39070.0	39670.0	—	Unique to GPHLV
—	CDS_39654_40986	—	—	39654.0	40986.0	—	Unique to GPHLV
—	CDS_40897_41965	—	—	40897.0	41965.0	—	Unique to GPHLV
—	CDS_41837_42860	—	—	41837.0	42860.0	—	Unique to GPHLV
—	CDS_42858_43824	—	—	42858.0	43824.0	—	Unique to GPHLV
—	CDS_43792_44263	—	—	43792.0	44263.0	—	Unique to GPHLV
—	CDS_44147_45446	—	—	44147.0	45446.0	—	Unique to GPHLV
—	CDS_45458_46907	—	—	45458.0	46907.0	—	Unique to GPHLV
—	CDS_46861_47194	—	—	46861.0	47194.0	—	Unique to GPHLV
—	CDS_47252_48377	—	—	47252.0	48377.0	—	Unique to GPHLV
—	CDS_48482_50375	—	—	48482.0	50375.0	—	Unique to GPHLV
—	CDS_50366_51188	—	—	50366.0	51188.0	—	Unique to GPHLV
—	CDS_51126_52863	—	—	51126.0	52863.0	—	Unique to GPHLV
—	CDS_52801_55165	—	—	52801.0	55165.0	—	Unique to GPHLV
—	CDS_55202_55778	—	—	55202.0	55778.0	—	Unique to GPHLV
—	CDS_55788_56547	—	—	55788.0	56547.0	—	Unique to GPHLV
—	ORF47	—	—	56548.0	56989.0	—	Unique to GPHLV

GPXV Protein/Gene	GPHLV Protein/Gene	GPXV Start	GPXV End	GPHLV Start	GPHLV End	% Identity	Notes
—	CDS_56548_56989	—	—	56548.0	56989.0	—	Unique to GPHLV
—	ORF50_1	—	—	58707.0	58749.0	—	Unique to GPHLV
—	CDS_58770_59661	—	—	58770.0	59661.0	—	Unique to GPHLV
—	RTA	—	—	59758.0	61558.0	—	Unique to GPHLV
—	G5	—	—	61706.0	61919.0	—	Unique to GPHLV
—	CDS_61706_61919	—	—	61706.0	61919.0	—	Unique to GPHLV
—	G4	—	—	62157.0	62460.0	—	Unique to GPHLV
—	CDS_62556_62931	—	—	62556.0	62931.0	—	Unique to GPHLV
—	CDS_63003_63738	—	—	63003.0	63738.0	—	Unique to GPHLV
—	CDS_63819_64758	—	—	63819.0	64758.0	—	Unique to GPHLV
—	CDS_64791_65439	—	—	64791.0	65439.0	—	Unique to GPHLV
—	of57 ex1	—	—	68045.0	68138.0	—	Unique to GPHLV
—	CDS_69881_70946	—	—	69881.0	70946.0	—	Unique to GPHLV
—	CDS_70948_72046	—	—	70948.0	72046.0	—	Unique to GPHLV
—	CDS_73095_75393	—	—	73095.0	75393.0	—	Unique to GPHLV
—	CDS_75392_76502	—	—	75392.0	76502.0	—	Unique to GPHLV
—	ORF67	—	—	88584.0	89367.0	—	Unique to GPHLV
—	ORF67a	—	—	89338.0	89623.0	—	Unique to GPHLV
—	CDS_91936_92065	—	—	91936.0	92065.0	—	Unique to GPHLV
—	CDS_92024_92132	—	—	92024.0	92132.0	—	Unique to GPHLV
—	K12 Positional Homolog	—	—	92412.0	92544.0	—	Unique to GPHLV

GPXV Protein/Gene	GPHLV Protein/Gene	GPXV Start	GPXV End	GPHLV Start	GPHLV End	% Identity	Notes
—	G2.1	—	—	92788.0	92899.0	—	Unique to GPHLV
—	G2	—	—	93445.0	93823.0	—	Unique to GPHLV
—	CDS_93653_93719	—	—	93653.0	93719.0	—	Unique to GPHLV
—	BCL2A1	—	—	94762.0	95293.0	—	Unique to GPHLV
—	G1	—	—	103242.0	103374.0	—	Unique to GPHLV
—	TR 1	—	—	103242.0	103374.0	—	Unique to GPHLV

Table S4. Comparative annotation of GPXV and GPHLV proteins.

This table presents a side-by-side comparison of predicted protein-coding genes in the Guinea Pig X Virus (GPXV) and Guinea Pig Herpes-Like Virus (GPHLV) genomes. For each GPXV gene or protein, the corresponding GPHLV homolog (if identified) is listed along with their genomic coordinates and percent identity. Notes indicate whether a gene is unique to GPXV or shows divergence from its GPHLV counterpart.
